# Machine learning prediction of moderate‐to‐severe acute kidney injury after ICU admission and cardiac surgery with urine trace elements

**DOI:** 10.1111/eci.70131

**Published:** 2025-10-03

**Authors:** Yang Chen, Ying Gue, Gregory Y. H. Lip, David S. Gardner, Mark A. J. Devonald

**Affiliations:** ^1^ Liverpool Centre for Cardiovascular Science, at University of Liverpool Liverpool John Moores University and Liverpool Heart & Chest Hospital Liverpool UK; ^2^ Department of Cardiovascular and Metabolic Medicine, Institute of Life Course and Medical Sciences University of Liverpool Liverpool UK; ^3^ Danish Center for Health Services Research, Department of Clinical Medicine Aalborg University Aalborg Denmark; ^4^ Medical University of Bialystok Bialystok Poland; ^5^ Faculty of Medicine and Health Sciences, School of Veterinary Medicine and Science University of Nottingham Nottingham UK; ^6^ Renal and Transplant Unit Liverpool University Hospitals NHS Foundation Trust Liverpool UK

**Keywords:** acute kidney injury, cardiac surgery, intensive care unit, machine learning, urinary trace elements

## Abstract

**Background:**

Acute kidney injury (AKI) is common and linked to poor outcomes, but early detection remains challenging. Previous research identified urinary trace elements (TE) as early AKI biomarkers in intensive care unit (ICU) or cardiac surgery patients. We aimed to explore whether urinary TE enhance machine learning (ML) models for AKI prediction.

**Methods:**

We constructed ML models using the ICU cohort. We filtered the variables and optimized hyperparameters before predicting Kidney Disease: Improving Global Outcomes stage 2–3 AKI using eight ML classifiers: light gradient boosting machine (LightGBM), random forest (RF), ML logistic regression, support vector machine, multilayer perceptron, eXtreme gradient boosting (XGBoost), Gaussian Naive Bayes and k‐nearest neighbors. External validation was performed in the cardiac surgery cohort.

**Results:**

Among 149 ICU patients (median age 56.0 [interquartile range (IQR): 43.5–67.0], 63.1% male), 25 developed stage 2–3 AKI; among 144 cardiac surgery patients (median age 70.0 [IQR: 62.0–76.0], 72.9% male), 12 developed stage 2–3 AKI. Each ML in the internal validation had area under the curve (AUC) above .7, with XGBoost having the highest (.813); LightGBM had the second highest AUC (.799), highest G‐mean (.567) and F1‐score (.545). In external validation, RF had the highest AUC (.740), XGBoost had the highest G‐mean (.289) and F1‐score (.286). Age, strontium and boron were consistently ranked among the top five most important features in LightGBM, RF and XGBoost.

**Conclusion:**

ML models primarily based on urinary TE can identify AKI risk in both clinical groups (ICU and cardiac surgery), with LightGBM, RF and XGBoost serving as high‐performance models for early prediction of stage 2–3 AKI.

## Graphical abstract

Acute kidney injury (AKI) is a frequent complication after ICU admission and cardiac surgery. In this study, 149 ICU patients and 144 post–cardiac surgery patients were evaluated using urinary trace elements with eight machine learning (ML) algorithms. LightGBM, Random Forest and XGBoost showed the highest predictive performance (AUCs .71–.85). Key predictive features included age, vanadium, boron, copper, molybdenum, strontium, albumin, zinc, phosphorus and lithium. ML models incorporating urinary trace elements may enable early stratification of moderate‐to‐severe AKI risk.

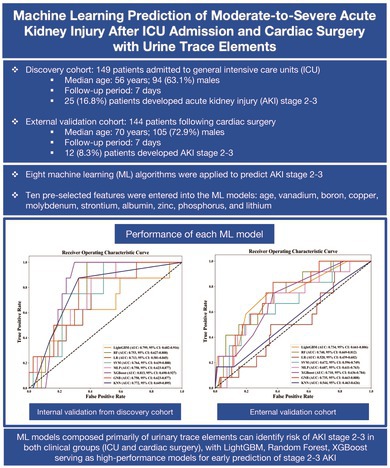

## 1 INTRODUCTIONS

Acute kidney injury (AKI) is common in hospitalized patients and is associated with poor outcomes including increased mortality and development of chronic kidney disease (CKD) and is very costly.^1^, ^2^, ^3^, ^4^ For decades, detection of AKI has relied on an increase in serum creatinine or a decrease in urine output, both of which are poor markers of kidney injury and can lead to delayed detection of AKI, which is likely to contribute to poor outcomes. There is increasing interest in the discovery and development of biomarkers for much earlier (within a few hours) detection of AKI, as there is evidence that earlier simple interventions, such as implementation of clinical care bundles, can improve outcomes.^5^

We previously identified urinary trace elements as early biomarkers of AKI in a porcine model of ischaemia‐reperfusion AKI and subsequently demonstrated, in observational clinical studies, that urinary copper, zinc and cadmium are biomarkers for early detection of AKI in adults admitted to the intensive care unit (ICU) or undergoing cardiac surgery.^6^

Machine learning (ML) has the potential to discover complex patterns and associations that are difficult to detect by traditional methods,^7^ and has powerful performance in disease or clinical outcome prediction.^8^, ^9^, ^10^, ^11^, ^12^, ^13^, ^14^ In this study, we investigated whether incorporation of urinary trace element data, from our previous observational cohort studies, into ML models, improved the performance of these biomarkers in the early detection of AKI.

## 2 METHODS

### 2.1 Study participants and inclusion criteria

**FIGURE 1:**
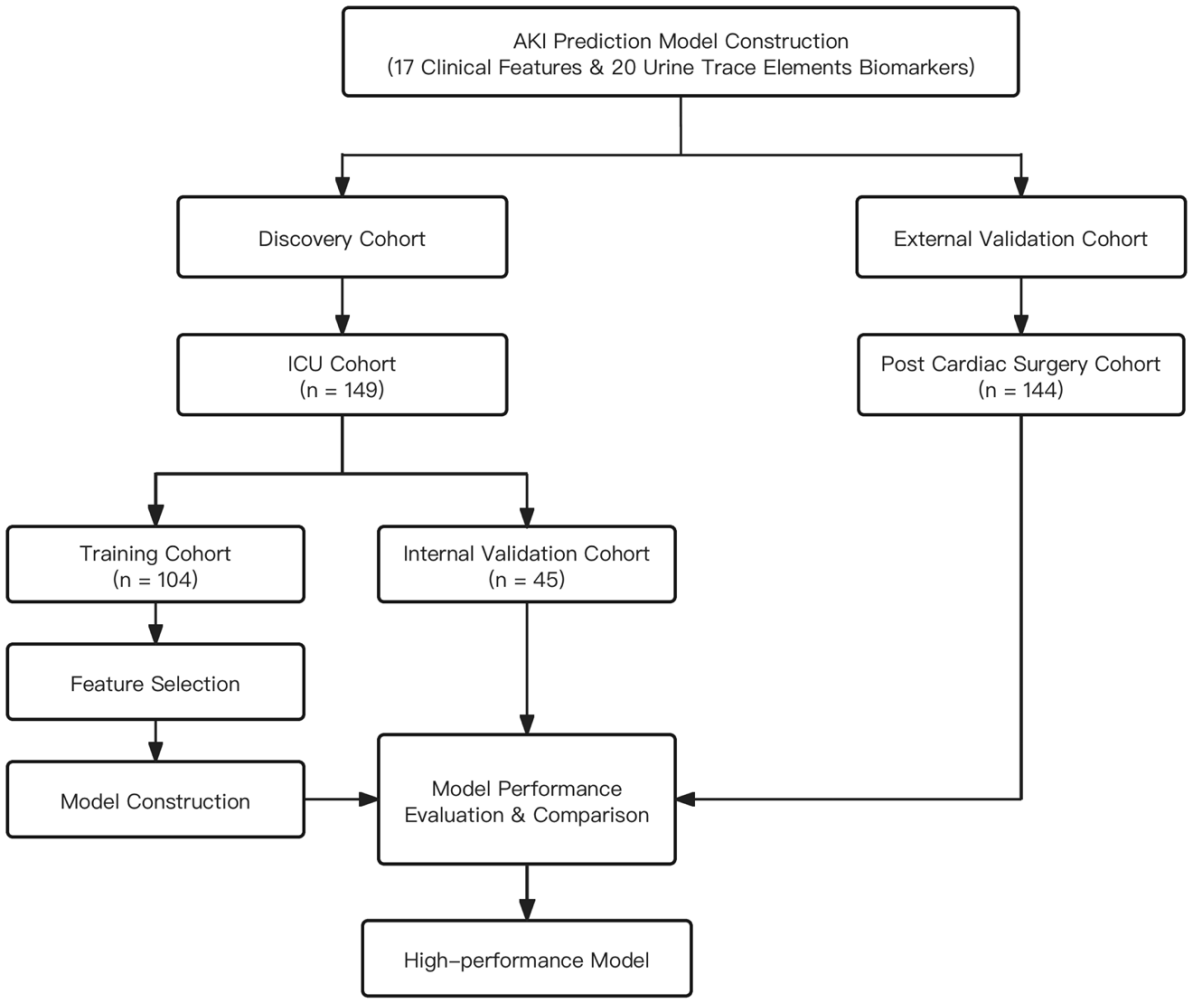
Flow chart of this analysis. AKI, acute kidney injury; ICU, intensive care unit.

Details of the discovery and validation cohorts are published.^6^ The discovery cohort (ethical approval number: 15/EM/0452) was recruited between October 2015 and February 2017, with inclusion criteria of (1) admission to general adult ICU, (2) age ≥17 years, (3) requirement for urinary catheter. Our external validation cohort (ethical approval number: 15/EM/0451) was recruited between October 2015 and December 2017, with inclusion criteria of (1) admission to the Trent Cardiac Centre for emergency or elective cardiac surgery, including coronary artery bypass graft surgery (CABG) (on‐pump), CABG (off‐pump), valve surgery, combined CABG/valve surgery and other procedures, (2) age ≥17 years, (3) >1 risk factor for AKI (according to National Institute for Health and Care Excellence Clinical Guideline 169).^15^ A flow chart summarising construction of the prediction model is shown in Figure 1. This study was conducted in accordance with the TRIPOD (Transparent Reporting of a multivariable prediction model for Individual Prognosis Or Diagnosis) guidelines, and the completed TRIPOD checklist is provided in the Appendix S1.

### 2.2 AKI definition

AKI is currently universally defined by Kidney Disease Improving Global Outcomes (KDIGO) criteria, which are based on either an increase in serum creatinine (sCr) or a fall in urine output (UO). In practice, sCr criteria are used far more widely than UO criteria because, for the latter, accurate measurement of hourly urine volume is required, which happens rarely outside ICU and renal units. Furthermore, KDIGO UO criteria are not explicit about how UO should be defined.^16^ We therefore used only KDIGO sCr criteria for our analyses.^17^ Baseline sCr was determined by the following criteria: (1) when sCr was available within 7 days of ICU admission or post‐cardiac surgery, the lowest value was used; (2) when sCr was available within 365 days but not within 7 days, the median of the sCr within 365 days was used; (3) where baseline sCr was unavailable, we imputed it using the Modification of Diet in Renal Disease (MDRD) equation,^18^, ^19^ assuming an estimated glomerular filtration rate (eGFR) of 75 mL/min/1.73 m^2^.^20^ Post‐admission (for ICU) or post‐operative (for cardiac surgery) sCr results, measured at least daily, were used to determine the incidence and KDIGO stage of AKI. If two or more episodes of AKI occurred during the study period, only the more or most severe AKI was used.

### 2.3 Data collection and outcomes

The primary outcome of interest was moderate‐to‐severe AKI (stage 2–3 AKI) within 7 days after ICU admission or cardiac surgery. In addition, we collected demographic characteristics, lifestyle, nephrotoxic medications, comorbidities and the first value of urine TE within 24 h after ICU admission or cardiac surgery. TEs were measured in 500 μL urine aliquots by inductively coupled plasma mass spectrometry (ICP‐MS, iCAP Q, Thermo Fisher Scientific Inc., Waltham, MA), using methods described previously.^6^

### 2.4 Feature selection and model construction

We randomly divided our discovery cohort into a training group and an internal validation cohort in a 7:3 ratio. Due to the limited sample size of our cohort, it was necessary to reduce the number of features entering the model to reduce the risk of overfitting. Therefore, we used three methods of feature selection: random forest (RF), light gradient boosting machine (LightGBM) and Boruta. Each method selected its corresponding top 10 important features, and the features selected by at least two of these methods simultaneously were used for model construction. In addition, we performed Pearson correlation analyses and calculated variance inflation factors to ensure that there was no multicollinearity among the variables in the model. We then imported these features into eight commonly used ML classifiers to predict stage 2–3 AKI, including LightGBM, RF, ML logistic regression (LR), support vector machines (SVM), multilayer perceptron (MLP), eXtreme gradient boosting (XGBoost), Gaussian Naive Bayes (GNB) and k‐nearest neighbors (KNN). The proportion of stage 2–3 AKI in our discovery cohort was about 17%, and to address the imbalance problem, we set class weights when constructing the model. The optimum hyperparameters for the MLs were obtained by random search combined with manual fine‐tuning and five‐fold cross‐validation.

### 2.5 Evaluation of parameters and model interpretation

First, we obtained the receiver operating characteristic curve (ROC) for each ML and calculated the area under the receiver operating characteristic curve (AUC) and 95% confidence intervals (CI). We calculated several metrics (including accuracy, specificity, precision, recall, F1‐score and G‐mean) for each classifier to assess their performance in the training, internal and external validation cohorts. Subsequently, we comprehensively considered the performance of each classifier in both internal and external validation to select high‐performing ML tools. Finally, we calculated the SHapley Additive exPlanations (SHAP) value for each feature within the high‐performing models to account for the contribution of each feature to the stage 2–3 AKI prediction. In addition, to further assess the ability of the model to discriminate between AKI stage 0 and AKI stage 2/3, we performed additional model evaluations in the ICU cohort and the post‐cardiac surgery cohort, respectively, with both removing AKI stage 1.

### 2.6 Web‐based prediction platform

To make our model practical, we also developed a model‐based web‐based prediction platform allowing clinicians to input features easily to determine the stage 2–3 AKI risk for ICU‐admitted or post‐cardiac surgery patients.

### 2.7 Statistical analysis

The data used for the present analysis did not contain any missing values, excluding baseline serum creatinine, which was handled separately as described above. The continuous variables in this study were nonnormally distributed; thus, we used the median with interquartile range (IQR) and the Mann–Whitney *U* test to compare differences between the stage 2–3 AKI group and non‐AKI/stage 1 AKI group. We used counts and percentages to describe categorical variables and Fisher's exact test or chi‐square test to compare differences between the two groups. All statistical significance levels were set at two‐tailed *p* < .05. In this study, we performed all analyses with SPSS software (version 29.0.0.1, IBM Corporation, Armonk, New York, United States) and Python (version 3.11.4, Python Software Foundation, Beaverton, Oregon, United States).

## 3 RESULTS

### 3.1 Patient characteristics

In the discovery cohort, median age was 56.0 years (IQR: 43.5 to 67.0), and 94 (63.1%) were male (Table 1). Twenty‐five (16.8%) patients developed stage 2–3 AKI. Compared with the non‐AKI and stage 1 AKI groups, the stage 2–3 AKI group was older (median: 62.0 vs. 55.0 years), had more diabetes (40.0% vs. 19.4%), sepsis (44.0% vs. 23.4%), hypertension (48.0% vs. 18.5%) and stroke (16.0% vs. 3.2%). For urine TEs, the stage 2–3 AKI group had higher copper concentration but lower boron, sodium, lithium, rubidium, strontium, molybdenum and caesium.

**TABLE 1:** Characteristics of patients in the ICU discovery cohort.

Variables	All	Non‐AKI and Stage 1 AKI	Stage 2–3 AKI	*P*
*N*	149	124	25	
Age, years	56.00 (43.50, 67.00)	55.00 (43.00, 66.00)	62.00 (49.00, 73.50)	.095
Male, *n* (%)	94 (63.1)	76 (61.3)	18 (72.0)	.369
Smoke, *n* (%)				
Never smoke	81 (54.4)	66 (53.2)	15 (60.0)	.709
Ever smoke	26 (17.4)	23 (18.5)	3 (12.0)	
Current smoke	42 (28.2)	35 (28.2)	7 (28.0)	
Nephrotoxic medications, *n* (%)	39 (26.2)	30 (24.2)	9 (36.0)	.223
Comobidities, *n* (%)				
Chronic kidney disaese	8 (5.4)	6 (4.8)	2 (8.0)	.622
Congestive heart failure	5 (3.4)	4 (3.2)	1 (4.0)	.845
Liver disease	13 (8.7)	11 (8.9)	2 (8.0)	.888
Diabetes	34 (22.8)	24 (19.4)	10 (40.0)	.036
Previous AKI	5 (3.4%)	4 (3.2)	1 (4.0)	.845
Urological obstruction	5 (3.4%)	4 (3.2)	1 (4.0)	.845
Sepsis	40 (26.8)	29 (23.4)	11 (44.0)	.047
COPD	8 (5.4)	7 (5.6)	1 (4.0)	.739
Hypertension	35 (18.5)	23 (18.5)	12 (48.0)	.003
Atrial fibrillation	3 (2.0)	2 (1.6)	1 (4.0)	.426
Peripheral vascular disease	1 (.7)	1 (.8)	0 (.0)	.652
Stroke	8 (5.4)	4 (3.2)	4 (16.0)	.027
Malignancy	27 (18.1)	22 (17.7)	5 (20.0)	.779
Urine trace elements				
Boron, mg/L	.35 (.17, .71)	.40 (.19, .78)	.14 (.09, .38)	<.001
Sodium, mg/L	830.75 (362.79, 1681.67)	902.61 (418.70, 1704.34)	605.41 (211.29, 1110.47)	.046
Magnesium, mg/L	22.78 (8.90, 52.12)	23.31 (9.95, 57.68)	17.39 (4.88, 41.81)	.125
Phosphorus, mg/L	486.82 (206.61, 955.57)	506.19 (210.82, 967.96)	330.31 (120.87, 867.53)	.192
Sulphur, mg/L	565.59 (315.70, 1059.08)	592.07 (312.19, 1065.58)	487.67 (319.06, 1041.09)	.847
Lithium, μg/L	7.18 (3.78, 14.71)	7.45 (3.92, 15.72)	4.59 (1.57, 9.25)	.005
Vanadium, μg/L	.55 (.30, 1.03)	.58 (.32, 1.04)	.45 (.16, .95)	.125
Chromium, μg/L	2.38 (1.07, 5.20)	2.23 (1.08, 5.34)	2.62 (.67, 4.26)	.446
Cobalt, μg/L	.33 (.16, .61)	.34 (.19, .67)	.25 (.14, .39)	.115
Copper, μg/L	18.60 (7.29, 50.49)	16.15 (5.93, 43.52)	39.17 (19.28, 92.90)	.003
Zinc, μg/L	1107.90 (448.51, 2043.75)	969.04 (435.51, 1910.36)	1504.70 (548.95, 3125.59)	.078
Arsenic, μg/L	5.83 (2.50, 25.53)	5.58 (2.40, 27.36)	6.70 (2.79, 17.11)	.764
Selenium, μg/L	16.52 (8.16, 34.28)	16.57 (8.10, 33.69)	14.19 (8.72, 44.21)	.477
Rubidium, μg/L	1166.37 (598.68, 1707.53)	1203.63 (628.95, 1820.18)	971.32 (454.69, 1286.22)	.025
Strontium, μg/L	36.85 (13.42, 80.80)	46.81 (18.57, 86.57)	10.28 (4.84, 45.30)	<.001
Molybedenum, μg/L	8.49 (3.00, 22.19)	10.64 (3.79, 25.20)	2.03 (1.54, 8.56)	.002
Caesium, μg/L	3.78 (2.21, 6.94)	4.32 (2.44, 7.24)	2.26 (1.31, 3.82)	.004
Barium, μg/L	4.20 (2.10, 8.97)	4.17 (1.86, 8.42)	4.42 (2.11, 9.57)	.907
Creatinine, μmol/L	5060.83 (2847.70, 9038.57)	5262.48 (2881.47, 9675.59)	4488.35 (2546.93, 7761.95)	.337
Albumin, g/L	.20 (.11, .42)	.19 (.11, .39)	.28 (.09, .55)	.381

Abbreviations: AKI, acute kidney injury; COPD, chronic obstructive pulmonary disease; ICU, intensive care unit.

### 3.2 Feature selection

We randomly divided the discovery cohort into a training cohort and an internal validation cohort in a 7:3 ratio. In the training cohort, we finally chose 10 variables for constructing the predictive model, including age, vanadium, boron, copper, molybdenum, strontium, albumin, zinc, phosphorus and lithium (Figure S1). In Figure S2, the coefficients between all variables were less than .7, and variance inflation factor values were less than 5.0, which implied that these selected features did not have strong correlation or multicollinearity.

### 3.3 Model Construction and Evaluation

**FIGURE 2:**
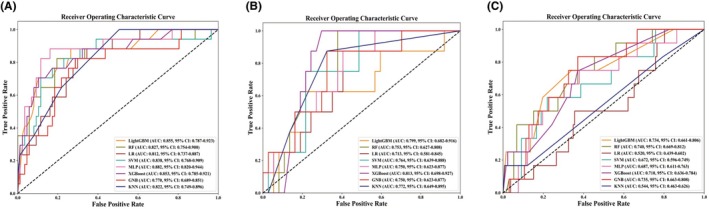
Receiver operating characteristic curves for eight machine learning classifiers in the training cohort (A), internal validation cohort (B) and external validation cohort (C). AUC, area under curve; CI, confidence interval; GNB, Gaussian Naive Bayes; KNN, k‐nearest neighbors; LightGBM, light gradient boosting machine; LR, logistic regression; MLP, multilayer perceptron; RF, random forest; SVM, support vector machine; XGBoost, eXtreme gradient boosting.

We imported the 10 selected features into eight ML classifiers, and the best hyperparameters of each classifier were obtained in the training cohort (Table S1). Based on Figure 2, the ROCs and AUCs for the training, internal validation and external validation cohorts indicated that there was no significant overfitting in each ML model.

In the internal validation cohort (Figure 2B), the AUCs of all MLs exceeded .7, with XGBoost obtaining the highest AUC (.813, 95% CI .698–.927) and ML LR the lowest AUC (.713, 95% CI .581–.845). When calculating the other metrics for MLs (Table 2), LightGBM had the highest F1 score (.545), recall (.750) and the second‐highest AUC (.799, 95% CI .682–.916); thus, LightGBM was considered the best performer in the internal validation cohort.

**TABLE 2:** The performance of classifiers in the internal validation cohort and external validation cohort.

Classifiers	AUC (95% CI)	Accuracy	Specificity	Precision	Recall	F1‐score	G‐mean
Internal validation cohort							
LightGBM	.799 (.682, .916)	.778	.784	.429	.750	.545	.567
Random forest	.753 (.627, .879)	.756	.811	.364	.500	.421	.427
Logistic regression	.713 (.581, .845)	.644	.649	.278	.625	.385	.417
Support vector machine	.764 (.639, .888)	.711	.784	.273	.375	.316	.320
Multilayer perceptron	.750 (.623, .877)	.800	.919	.400	.250	.308	.316
XGBoost	.813 (.698, .927)	.778	.838	.400	.500	.444	.447
Gaussian Naive Bayes	.750 (.623, .877)	.756	.865	.286	.250	.267	.267
K‐nearest neighbours	.772 (.649, .895)	.800	.973	.000	.000	.000	.000
External validation cohort							
LightGBM	.734 (.661, .806)	.819	.871	.150	.250	.187	.194
Random forest	.740 (.669, .812)	.736	.758	.158	.500	.240	.281
Logistic regression	.520 (.439, .602)	.181	.106	.092	1.000	.169	.303
Support vector machine	.672 (.596, .749)	.479	.462	.101	.667	.176	.260
Multilayer perceptron	.687 (.611, .763)	.910	.992	.000	.000	.000	.000
XGBoost	.710 (.636, .784)	.896	.955	.333	.250	.286	.289
Gaussian Naive Bayes	.735 (.663, .808)	.896	.970	.200	.080	.118	.126
K‐nearest neighbours	.544 (.463, .626)	.917	1.000	.000	.000	.000	.000

Abbreviations: AUC, area under curve; CI, confidence interval; LightGBM, light gradient boosting machine; XGBoost, eXtreme gradient boosting.

Based on the prior feature selection, the characteristics of the external validation cohort are shown in Table S2. In the external validation cohort (Figure 2C), only AUCs of LightGBM, RF, XGBoost and GNB exceeded .7, with RF having the highest AUC (.740, 95% CI .669–.812) and ML LR having the lowest AUC (.554, 95% CI .549–.560). Further calculating other metrics for MLs with AUCs over .7 (Table 2 and Figure S3), GNB had the lowest G‐mean (.126) and F1‐score (.118) and was considered to have the weakest generalisation ability. XGBoost had some other strengths with the highest G‐mean (.289) and F1‐score (.286). Therefore, LightGBM, RF and XGBoost were retained as acceptable high‐performance MLs for early prediction of stage 2–3 AKI in ICU and cardiac surgery patients.

After removing stage 1 AKI from the ICU cohort and post‐cardiac surgery cohort, LightGBM, RF and XGBoost identified slightly better performance for distinguishing stage 0 and stage 2/3 AKI (Table S3). In the ICU cohort, XGBoost had the highest AUC (.868, 95% CI .809–.927) and F1‐score (.588); LightGBM had the highest G‐mean (.601). In the post‐cardiac surgery cohort, RF had the highest AUC (.753, 95% CI .676–.830) and XGBoost had the highest F1‐score (.316) and G‐mean (.327).

### 3.4 Feature importance

**FIGURE 3:**
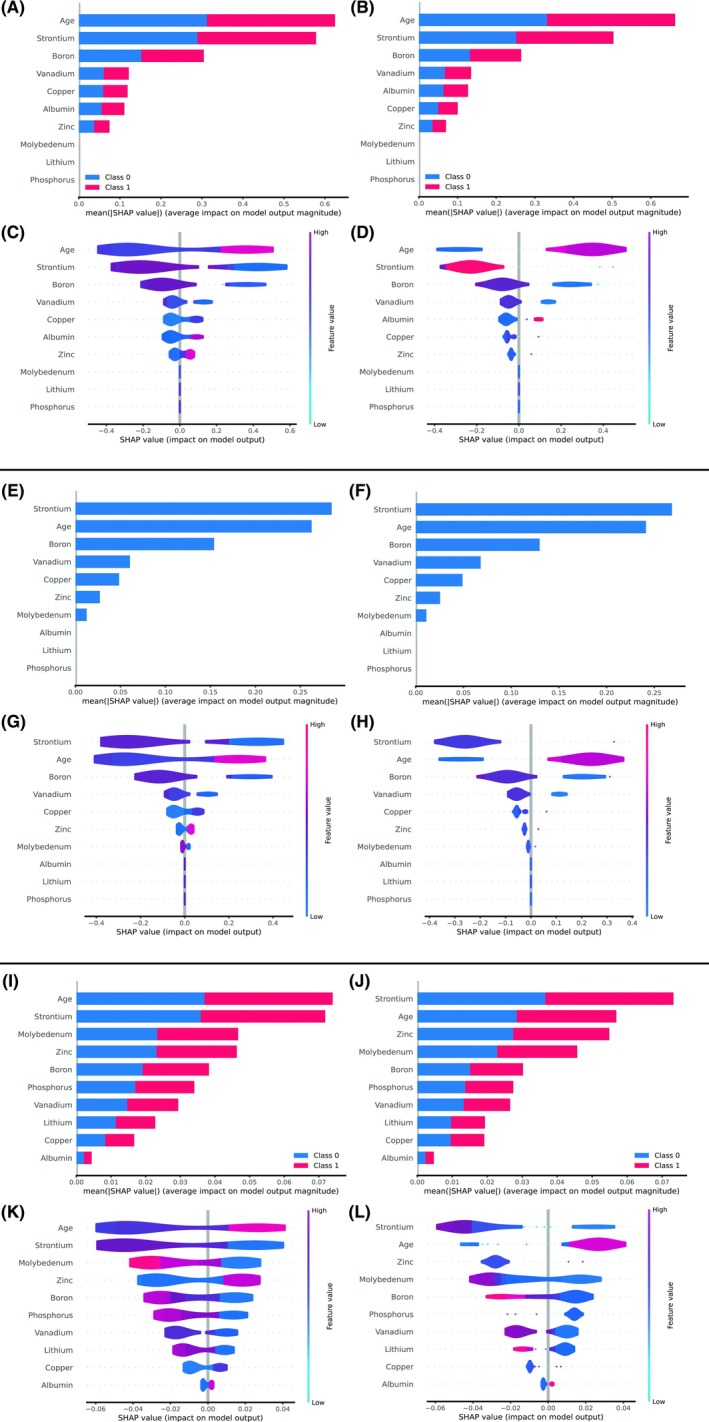
SHAP value and importance of each feature in our LightGBM (A and C from internal validation; B and D from external validation), XGBoost (E and G from internal validation; F and H from external validation) and random forest (I and K from internal validation; J and L from external validation). XGBoost, LightGBM, light gradient boosting machine; SHAP, SHapley Additive exPlanation.

To explain further the contribution of each feature to the prediction of stage 2–3 AKI, we calculated and ranked the SHAP values of features in LightGBM, RF and XGBoost. Based on Figure 3A–L, the top five ranked features in LightGBM, RF and XGBoost were partially different, with common features including age, strontium and boron.

### 3.5 Online Prediction Platform

Based on our LightGBM, RF and XGBoost data, we constructed a simple and practical web‐based platform (http://162.62.58.247:8080/static/index.html) outputting probabilities for assessing stage 2–3 AKI risk by inputting appropriate parameters. Figure S4 illustrates the use of this online platform; in this example, the patient is 65 years old, the corresponding values of urine TE are entered, and then the predicted probability of developing stage 2–3 AKI as derived by LightGBM is .63.

## 4 DISCUSSION

In our study, we identified 1 clinical feature and 9 urine TEs associated with early identification of stage 2–3 AKI by ML. Secondly, we developed LightGBM, RF and XGBoost models which performed well at identifying stage 2–3 AKI in both ICU and cardiac surgery patients.

To our knowledge, this study is the first to show that MLs composed mainly of urine TEs are effective in predicting AKI in ICU and cardiac surgery patients. This provides new insights into the future adoption of urine TEs into clinical practice or into other predictive models that may be valuable for clinical decision‐making and risk stratification in AKI.

In our previous study, cadmium, copper and zinc were shown to be effective markers for early identification of stage 2–3 AKI.^6^ However, in the current study, cadmium was not selected into the model. The reason for the apparent difference in performance of cadmium might relate to the fact that, in the original study, urine TEs were measured at multiple time points as independent samples to explore the association between urine TEs and AKI, which improved the statistical efficacy and smoothed the ROC curves. However, TE data at different time points for the same patient are not completely independent, which may lead to overestimation of the predictive performance or data redundancy. To address these potential problems from the original study, for this study we selected only the first urine TE result within 24 h after ICU admission or cardiac surgery, to explore the performance of urine TEs in predicting stage 2–3 AKI by ML. Urine copper and zinc were again demonstrated to be associated with the development of stage 2–3 AKI, whereas urine cadmium was not selected by ML. This may be due to limited sample size and randomly splitting the cohort. Therefore, interpretation of the current results still requires caution, and further validation of the results of the present study is necessary, in a larger sample.

How do our ML models compare with other prediction tools for AKI in ICU or cardiac surgery adult cohorts? Schwager et al. constructed an XGBoost model for the prediction of stage 2–3 AKI using commonly used ICU parameters, with AUCs of .715 for the internal validation cohort.^21^ In our study, AUCs of models in internal validation were higher, providing evidence that urine TEs may be valuable biomarkers for predicting AKI. However, there is a need to explore further whether urine TEs combined with ICU parameters can achieve more accurate predictive models. Lee et al. constructed ML models to predict cardiac surgery‐associated AKI using 39 features and, in the test cohort, XGBoost predicted stage 2–3 AKI with AUC .74 and 95% CI .70–.79.^22^ AUCs of LightGBM, RF and XGBoost in our external validation set were similar to the performance of their best model, but our model relied on only 10 features and demonstrated a significant advantage in terms of clinical utility. Also, their model was not externally validated.

Given that urinary TEs have demonstrated effectiveness in early detection of AKI in both ICU and cardiac surgery, they might also have the potential for broader clinical application in predicting AKI in other clinical groups at high risk of AKI, such as patients undergoing major non‐cardiac surgery, those with heart failure, or those receiving nephrotoxic drugs including chemotherapy.

We retained three high‐performing ML models, LightGBM, RF and XGBoost, and used SHAP plots to explain the relative importance of each feature in them. We found that the following three features were in the top 5 in each model: age, strontium and boron. Older age is recognized as a risk factor for AKI due to renal ageing and increased probability of comorbidities and toxicity from drug use.^23^ Age was also identified as an important predictive feature in other ML models for predicting AKI.^24^, ^25^

There have been previous studies reporting an association between urinary TEs and kidney function, but prior to our published study, there have been no reports of urinary TEs as biomarkers for early detection of AKI. Yang et al. investigated the associations of 23 plasma and urine metal levels with eGFR in a rural Chinese cohort and showed that higher levels of urinary strontium were significantly associated with a lower risk of abnormal renal function (Q1 served as the reference group; Q2: odds ratio [OR] .38, 95% CI .23–.65; Q3: OR .24, 95% CI .12–.45; Q4: OR .08, 95% CI .03–.21; all *p* < .001), the highest level of urinary rubidium was significantly associated with a low risk of abnormal renal function (OR .16, 95% CI .07–.37, *p* < .001), whereas the highest level of urinary copper was significantly associated with a high risk of abnormal renal function (OR 3.70, 95% CI 1.92–7.14, *p* < .001).^26^ Our results showed lower urine strontium and rubidium and higher urine copper in the stage 2–3 AKI group compared with the non‐AKI or stage 1 AKI group, similar to theirs, but urinary rubidium was not selected into our model, which may be due to our limited sample size or possibly ethnic or environmental heterogeneity. There is limited evidence for the association of urinary boron with abnormal kidney function. Pahl et al. reported that renal fractional excretion of boron in non‐pregnant females was 43.2 ± 21.5%, suggesting that boron is significantly reabsorbed in renal tubules and that urinary boron is strongly associated with renal function.^27^ Kremer et al. evaluated the association between urinary boron and eGFR in renal transplant recipients; multifactorial linear regression demonstrated significant positive correlation between log_2_ urine boron excretion and eGFR (*Standard β* = .15, 95% CI .08–.23).^28^

### 4.1 Limitations

Our study provides further evidence for urinary TEs as noninvasive biomarkers for early detection of AKI and suggests that ML methods might enhance the performance of these biomarkers. Several important limitations of our study must be highlighted. First, although our LightGBM, RF and XGBoost models demonstrated good performance in internal and external validation cohorts, sample sizes for both our ICU and cardiac cohorts were less than 200, so further validation in larger cohorts is needed to validate the performance of MLs more thoroughly. Second, due to the unbalanced nature of the ICU and cardiac surgery cohorts, LightGBM, RF and XGBoost should be interpreted with caution, even though they showed good differentiation for the minority class of stage 2–3 AKI. Third, since the feature selection was based on the training cohort with limited sample size, we restricted the number of features entering the model, which may have led to the omission of potentially predictive features. Fourth, we did not include risk factors reported in previous studies related to ICU and cardiac surgery associated AKI, such as ICU severity scores, intraoperative urine output and echocardiographic parameters, which may adversely affect AUCs of our MLs. Fifth, our method of defining baseline sCr followed established practice, using available values or imputation via the MDRD equation when necessary. However, the use of a fixed eGFR assumption for imputation may not reflect individual kidney function and could lead to AKI misclassification in some cases. Future studies with more complete longitudinal renal data may help improve accuracy in baseline sCr estimation and AKI classification. Finally, our study was based on data from a single centre with a predominantly white British population, so it is unclear whether the results are generalisable to other regions or ethnicities.

## 5 CONCLUSION

Following our previous report demonstrating that urinary TEs are biomarkers for early detection of AKI in adults admitted to the ICU or undergoing cardiac surgery, we have now shown that urinary TEs incorporated into ML models also associate with the development of stage 2–3 AKI in these clinical groups. In this study, ML was not applied to select individual predictors of AKI, but rather to integrate a panel of 10 pre‐selected TEs and clinical variables to improve overall prediction of stage 2–3 AKI. The relative contribution of these features varied, with age, boron and strontium consistently ranking highly across models. Our data suggest that ML models incorporating urinary TEs might result in better biomarker performance than urinary TEs alone and that a wider range of urinary TEs might be clinically relevant (such as boron and strontium, in addition to previously reported copper, zinc and cadmium) when ML models are employed. As several of these urinary TEs are potentially easily and cheaply measurable as point‐of‐care tests, further validation is required in larger cohorts and further studies in other patient groups at risk of AKI are required.

## AUTHOR CONTRIBUTIONS

Yang Chen: conceptualisation, methodology, data acquisition and management, formal analysis, visualisation, writing the original draft and review and editing. Ying Gue: review and editing and validation. Gregory Y.H. Lip: methodology, review and editing and validation and supervision. David S. Gardner: conceptualisation, data acquisition and management, review and editing and project administration. Mark A.J. Devonald: conceptualisation, data acquisition and management, review and editing, project administration and supervision. All authors read and approved the final manuscript.

## CONFLICT OF INTEREST STATEMENT

Mark A. J. Devonald acknowledges funding support for the original clinical studies that generated the clinical data from the National Institute for Health Research Invention for Innovation (i4i) programme (II‐LB‐0216‐20008). Additionally, Mark A. J. Devonald is a co‐inventor (with David S. Gardner) on a patent granted by the United States Patent and Trademark Office and the European Patent Office titled “Biomarkers related to kidney function and methods involving their use” (4480/3695/P/GB). Mark A. J. Devonald also serves as the co‐chair of the Clinical Practice Guidelines Committee of the United Kingdom Kidney Association. David S. Gardner acknowledges funding support for the original clinical studies that generated the clinical data from the National Institute for Health Research Invention for Innovation (i4i) programme (II‐LB‐0216‐20008). Additionally, David S. Gardner is a co‐inventor (with Mark A. J. Devonald) on a patent granted by the United States Patent and Trademark Office and the European Patent Office titled “Biomarkers related to kidney function and methods involving their use” (4480/3695/P/GB). Other authors declare no conflicts of interest.

## Supporting information


Appendix S1.

## Data Availability

The data that support the findings of this study are available from the corresponding authors upon reasonable request.
