# Supplementary material for: Machine learning prediction of moderate‐to‐severe acute kidney injury after ICU admission and cardiac surgery with urine trace elements
Source: Eur J Clin Invest. 2025 Oct 3;56(1):e70131. doi: 10.1111/eci.70131 (PMC12817231; doi:10.1111/eci.70131)
Supplement: Supplementary file 1 — Appendix S1. [file ECI-56-e70131-s001.docx]

Supplementary Table S1. Best hyperparameters of each classifier.

| **Classifiers** | **Hyperparameters** | |
| --- | --- | --- |
| Light Gradient Boosting | boosting_type | ‘gbdt’ |
|  | objective | ‘binary’ |
|  | n_estimators | 120 |
|  | num_leaves | 30 |
|  | max_depth | 5 |
|  | learning_rate | 0.01 |
|  | min_child_weight | 1 |
|  | min_child_samples | 33 |
|  | class_weight | ‘balanced’ |
|  |  |  |
| Random Forest | n_estimators | 118 |
|  | max_depth | 5 |
|  | min_samples_leaf | 30 |
|  | min_samples_split | 2 |
|  | criterion | ‘gin’ |
|  | random_state | 42 |
|  | class_weight | ‘balanced’ |
|  |  |  |
| Logistic Regression | C | 0.1 |
|  | penalty | l2 |
|  | solver | 'lbfgs' |
|  | class_weight | 'balanced' |
|  |  |  |
| eXtreme Gradient Boosting | booster | ‘gbtree’ |
|  | objective | ‘binary: logistic’ |
|  | eta | 0.01 |
|  | max_depth | 4 |
|  | min_child_weight | 10 |
|  | n_estimators | 129 |
|  | scale_pos_weight | 3 |
|  |  |  |
| Gaussian Naive Bayes | var_smoothing | 1e-09 |
|  |  |  |
|  |  |  |
| Support Vector Machine | C | 1 |
|  | kernel | 'rbf' |
|  | probability | True |
|  | tol | 0.001 |
|  | gamma | 0.008 |
|  | class_weight | 'balanced' |
| Multilayer Perceptron | solver | 'adam' |
|  | activation | 'relu' |
|  | hidden_layer_sizes | [7,] |
|  | alpha | 0.0001 |
|  | random_state | 1 |
|  | max_iter | 500 |
|  |  |  |
| K-Nearest Neighbors | n_neighbors | 7 |
|  | P | 1 |
|  | weights | ‘uniform’ |

Supplementary Table S2. Characteristics of patients in the post cardiac surgery cohort.

| **Variables** | **All** | **Non-AKI & AKI Stage 1** | **AKI Stage 2/3** | ***P*** |
| --- | --- | --- | --- | --- |
| N | 144 | 132 | 12 |  |
| Age, years | 70.00 (62.00, 76.00) | 70.00 (62.00, 76.00) | 70.00 (62.25, 76.75) | 0.756 |
| Male, n (%) | 105 (72.9) | 96 (72.7) | 9 (75.0) | 0.865 |
| Urine Trace Elements |  |  |  |  |
| Boron, mg/L | 0.23 (0.13, 0.35) | 0.23 (0.13, 0.35) | 0.21 (0.13, 0.36) | 0.573 |
| Phosphorus, mg/L | 78.96 (43.23, 170.16) | 80.44 (42.22, 164.48) | 72.73 (62.28, 182.40) | 0.778 |
| Lithium, μg/L | 4.20 (3.00, 6.50) | 4.26 (3.04, 6.63) | 3.72 (2.79, 4.52) | 0.176 |
| Vanadium, μg/L | 0.48 (0.33, 0.72) | 0.48 (0.35, 0.75) | 0.32 (0.21, 0.52) | 0.013 |
| Copper, μg/L | 5.06 (2.50, 8.70) | 4.92 (2.50, 8.97) | 6.61 (2.17, 7.60) | 0.968 |
| Zinc, μg/L | 314.51 (171.92, 498.62) | 309.39 (169.22, 497.31) | 377.70 (204.73, 538.20) | 0.422 |
| Strontium, μg/L | 48.07 (35.63, 70.82) | 50.25 (36.66, 72.17) | 35.57 (23.17, 49.06) | 0.012 |
| Molybedenum, μg/L | 9.55 (5.54, 15.44) | 9.55 (5.54, 15.44) | 9.04 (5.28, 14.46) | 0.879 |
| Albumin, g/L | 0.10 (0.05, 0.21) | 0.10 (0.05, 0.21) | 0.14 (0.04, 0.23) | 0.775 |

Abbreviations: AKI, acute kidney injury.

Supplementary Table S3. The evaluation of three high-performance classifiers in the ICU and post-cardiac surgery cohort with AKI 0 stage and AKI 2/3 stage.

| **Classifiers** | **AUC (95% CI)** | **Accracy** | **Specificity** | **Precision** | **Recall** | **F1-score** | **G-mean** |
| --- | --- | --- | --- | --- | --- | --- | --- |
| ICU cohort |  |  |  |  |  |  |  |
| LightGBM | 0.860 (0.799, 0.920) | 0.787 | 0.794 | 0.475 | 0.760 | 0.585 | 0.601 |
| Random Forest | 0.823 (0.757, 0.889) | 0.819 | 0.873 | 0.536 | 0.600 | 0.566 | 0.567 |
| XGBoost | 0.868 (0.809, 0.927) | 0.835 | 0.892 | 0.577 | 0.600 | 0.588 | 0.588 |
| Post-cardiac Surgery cohort | |  |  |  |  |  |  |
| LightGBM | 0.734 (0.661, 0.806) | 0.819 | 0.871 | 0.150 | 0.250 | 0.187 | 0.194 |
| Random Forest | 0.753 (0.676, 0.830) | 0.733 | 0.759 | 0.188 | 0.500 | 0.273 | 0.307 |
| XGBoost | 0.711 (0.630, 0.792) | 0.892 | 0.963 | 0.429 | 0.250 | 0.316 | 0.327 |

Legend: AUC, area under curve; CI, confidence interval; LightGBM, light gradient boosting machine; XGBM, eXtreme gradient boosting machine.


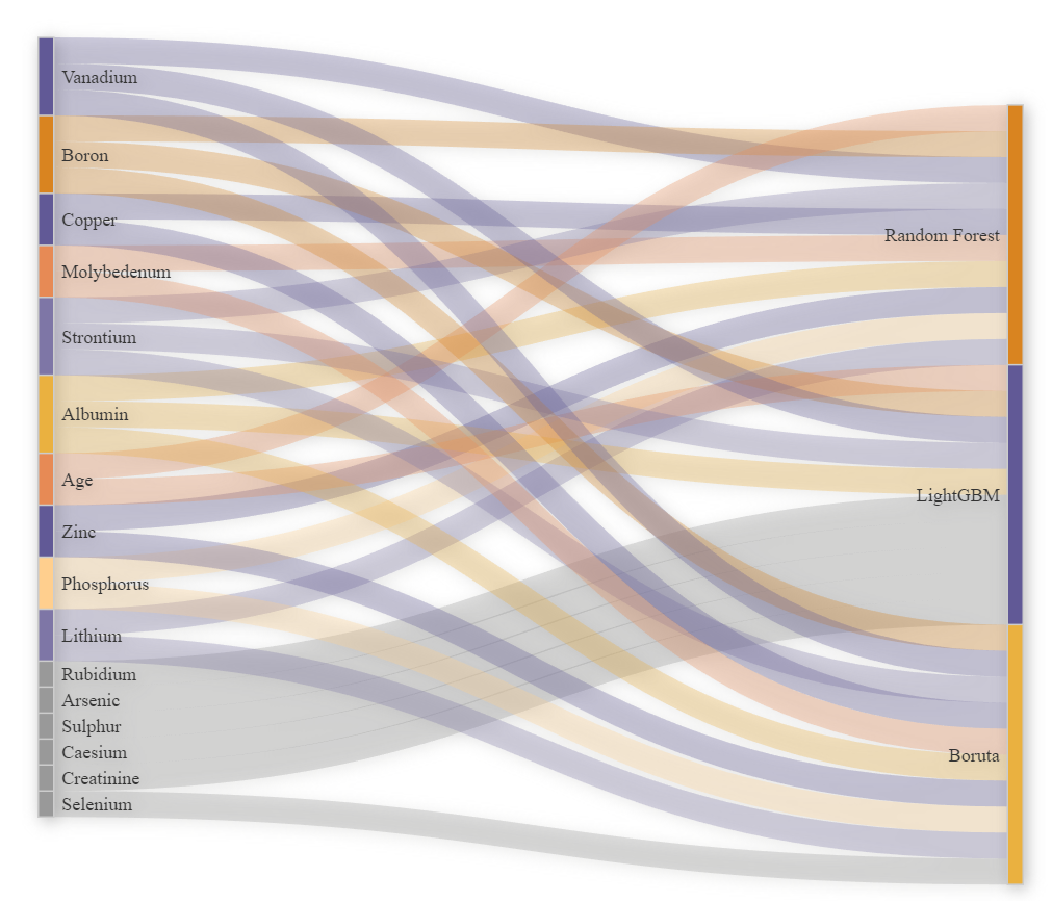


Supplementary Figure S1. Sankey diagrams for feature selection based on random forest, LightGBM and boruta (These three algorithms selected 16 features, based on the flow of Sankey diagrams, we finally selected 10 features entering the model [non-grey markers]). LightGBM, light gradient boosting machine.


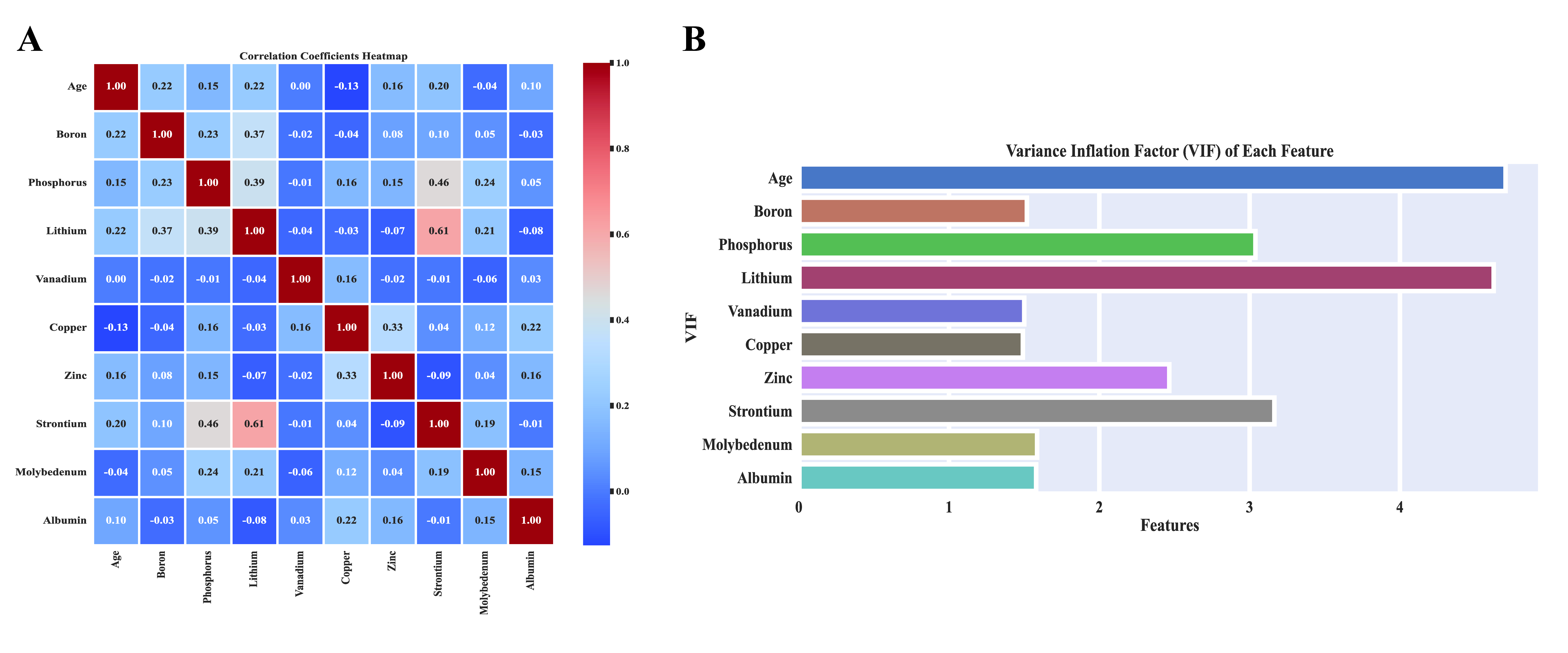


Supplementary Figure S2. Variables covariance test (A) and multicollinearity test (B) for the selected features.


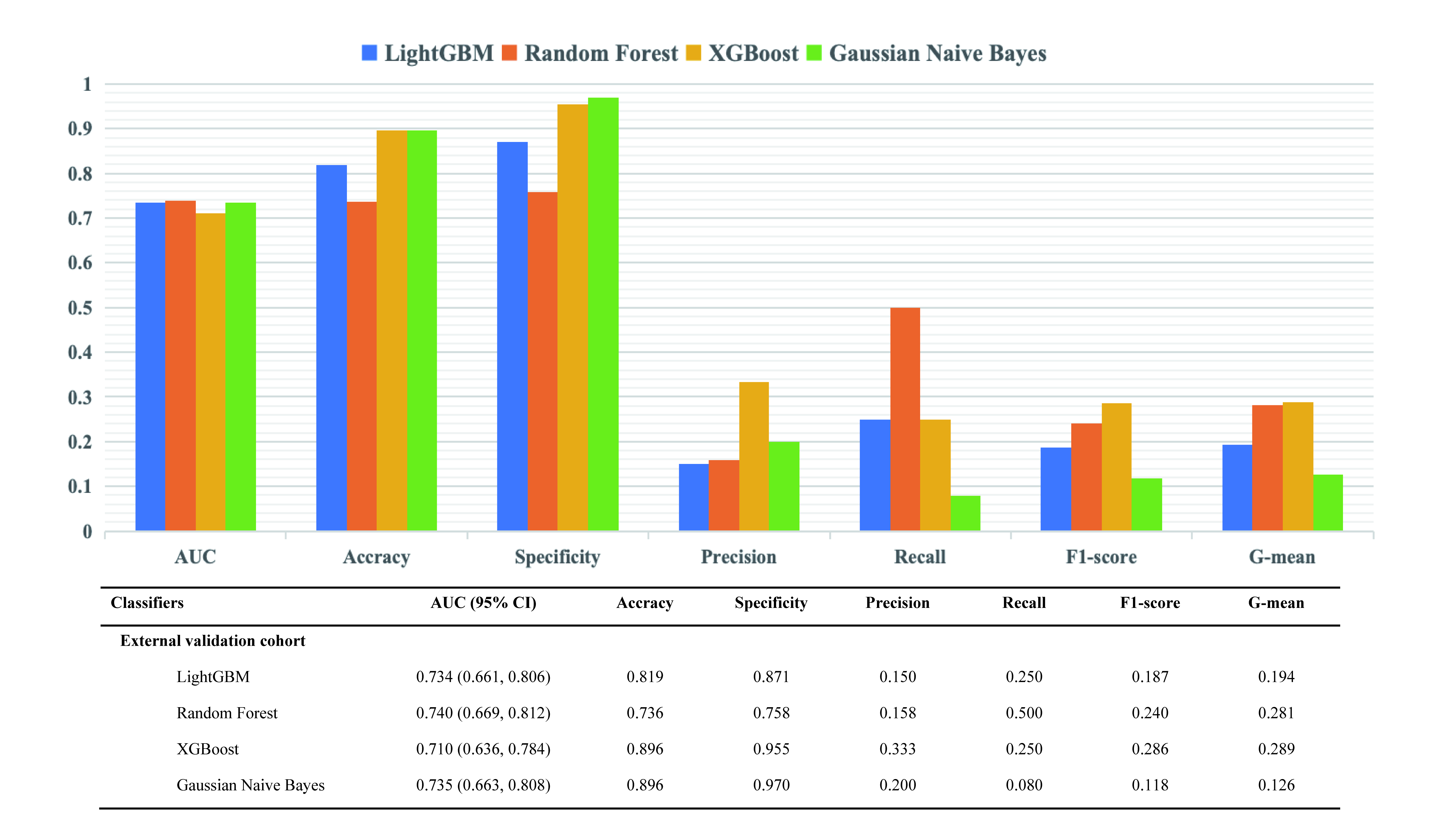


Supplementary Figure S3. Assessments of the four high-performance machine learning classifiers in the external validation cohort. AUC, area under curve; CI, confidence interval; LightGBM, light gradient boosting machine; XGBoost, eXtreme gradient boosting.


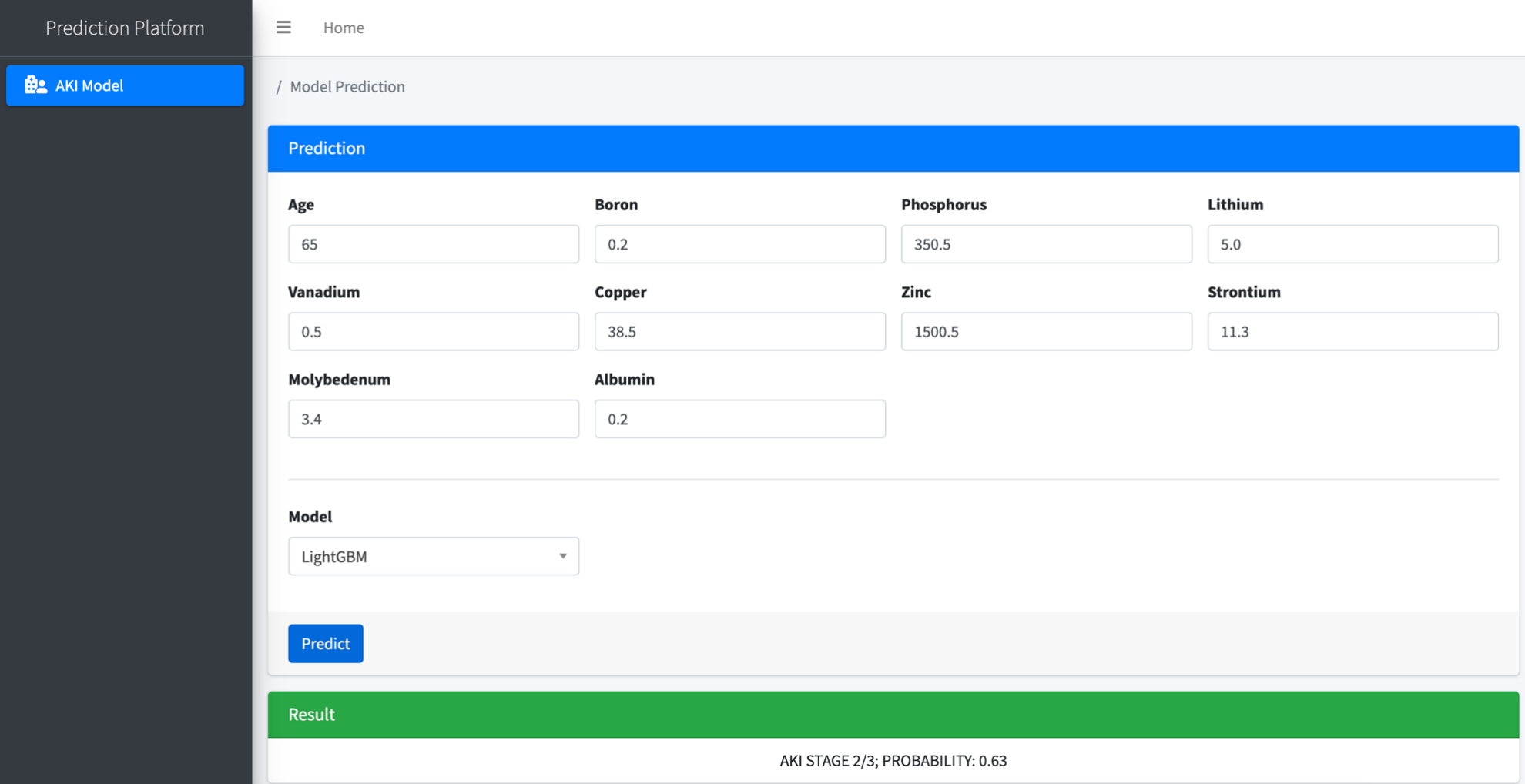


Supplementary Figure S4. The user interface of the website prediction platform. AKI, acute kidney injury.
